# Introducing a comprehensive high-stake online exam to final-year dental students during the COVID-19 pandemic and evaluation of its effectiveness

**DOI:** 10.1080/10872981.2020.1826861

**Published:** 2020-10-01

**Authors:** Khaled Khalaf, Mohamed El-Kishawi, Mohammad Adel Moufti, Sausan Al Kawas

**Affiliations:** College of Dental Medicine, University of Sharjah, Sharjah, UAE

**Keywords:** Online assessment, higher education, blackboard, dental education, computer-mediated assessment, summative assessment

## Abstract

**Background:**

Dental education involves teaching and assessing the acquisition of verifiable domains that require superior psychomotor, communication, and cognitive skills. Evolving technologies and methods of assessment could enhance student learning environment and improve tutor assessment experience.

**Objective:**

The aim of this study was to introduce the application of a comprehensive high-stakes online exam to final-year dental students during the COVID-19 pandemic and evaluate its effectiveness.

**Design:**

A high-stakes exam was introduced and implemented online to the final-year dental students prior to their graduation. The exam consisted of four components: MEQs, MCQs, OSCE and an oral exam. The exam and invigilation were conducted using Blackboard and MS Teams programs. Stakeholders’ views of the exam were obtained using two tailored surveys, one for students and another for faculty; both included closed- and open-ended questions.

**Results:**

The exam was run successfully without untoward events. Both students and staff were satisfied with the online exam with the latter being more satisfied than the former. Students with previous experience in online learning system were more satisfied with the online exam compared with those with less experience (*p* < 0.05). The main issues raised by students’ satisfaction with the exam were: inadequacy of time for the MEQ part, prevention of back tracking in the MCQ part and minor technological issues, whereas those raised by faculty members were increased time required to complete the exam setup and grading compared to the paper-based exam and minor technological issues.

**Conclusions:**

A newly introduced, multi-format, online high-stakes exam was implemented successfully to final-year dental students with minor technological issues and good satisfaction by students and staff alike.

## Introduction

Assessment is an essential component in the learning process because most students focus on assessment and consider it as a success indicator of their performance; hence, it has the power to drive students’ learning [1,2]. Various assessment methods have been discussed within the body of literature in dental education; however, the selection of assessment method depends on the purpose of its use and whether it is summative or formative or both. Summative assessment is outcome-dependent while formative assessment relates to in-process evaluation of students’ performance [3]. Different assessment tool criteria have been identified by previous studies including validity, reliability, educational impact, feasibility, and cost [4]. Nevertheless, a single assessment method will not assess all domains of students’ performance, and each method has its strengths and weaknesses. Thus, the use of various assessment methods is essential in order to compensate for the shortcomings of one method by the advantages of another and the choice should be dictated by fitness for purpose [5].

Assessment for learning is an educational concept that motivates both educator and learner to actively improve the learning process and facilitate a positive attitude towards future learning. Assessment in dental education should include a diagnostic component in order to identify learning barriers and student weaknesses [6,7].

Assessment of learning is more comprehensive in nature as it is undertaken at the end of the program to ensure that a student has reached a set of defined goals and objectives [8]. One of the purposes of assessment of learning is to decide whether a student has successfully achieved a learning outcome [9]. This could be used to identify a student’s competency of the skills learnt, knowledge and professional values required for beginning an independent dental practice. Other purposes of assessment include how much our students have retained of the required knowledge, how satisfied we are with what students have learned and the impact this may have on our future plans for teaching [10–12].

The modes of conventional coursework summative assessment used at the University of Sharjah, College of Dental Medicine (CDM) were the Objective Structured Clinical Examination (OSCE), Multiple Choice Questions (MCQ), Modified Essay Questions (MEQ), and oral examination. These exams are parts of the graduation exam (Exit exam) requirements for the degree of Bachelor of Dental Surgery (BDS) at the University of Sharjah and does not exist in other medical/dental colleges. The Exit exam has been developed and implemented at CDM, University of Sharjah several years prior to COVID-19 pandemic.

Considering the current Corona Virus (COVID-19) pandemic, closure of dental teaching and training facilities was enforced by the government bodies in many countries as a preventive measure [13]. This closure mandated the search for alternative effective and reliable online teaching and assessment tools [14]. Online learning and assessment have been developed and subsequently used widely over the last few years in higher education [15–17]. This was evident from the reported use of online resources and management systems for formative assessment (assessment for learning) and summative assessment (assessment of learning) forms [18].

Advancement in information technology gave rise to multipurpose computer-assisted educational assessment programs, which have transformed higher education fundamentally. Contemporary medical education witnessed a rapid change towards the web-based learning (WBL) to enhance the effectiveness of educational programs [19]. There are different types of WBL systems such as Computer-Mediated Communication (CMC) [20], Web Course Tools (WebCT) [21], Microsoft Teams (Teams) [22], and Blackboard (Bb) [23].

Teams is a cloud-based application and a digital hub used for conversations, virtual meetings, sharing files and applications in a single Learning Management System (LMS) [24]. Despite being in its earlier stage of development, Teams system has shown the potential to be an effective computer-supported collaborative learning process [22]. Blackboard is a web-based server software that provides virtual learning environment, assessment and course management system. It is a multimedia, curriculum-driven learning system that provides instructors with control and flexibility [25]. For example, various interactive learning tools such as announcement, calendar, tasks, assessment, grading, and user manual are available in Bb.

Blackboard has been in use at CDM, University of Sharjah for several years as means of communication with students, deployment of educational materials and carrying out some assessments; whereas MS Teams was introduced at the college post-COVID-19 pandemic as a forum to hold meetings and invigilation of exams. Bb training is usually given to students and academic staff at the beginning of, and throughout each academic year. It has been reported that opinions and attitudes of the users can affect any technology implementation [26]. Therefore, the effective utilization of the online assessment system depends mainly on students and faculty members’ background, readiness, and acceptance of such system.

There has been a lack of studies investigating different aspects of online assessment in higher education. Therefore, the purpose of this study was to introduce our experience of implementing a comprehensive high-stakes online exam to final-year dental students at college of dental medicine during the COVID-19 pandemic and evaluate its effectiveness in terms of absence of technological issues and good satisfaction by students and staff alike.

## Materials and methods

### Structure of the online assessment

The online assessment is called an Exit exam which is an important constituent of the dental education/training programme to measure and provide feedback on the student’s knowledge, clinical skills, attitudes, professional qualities, and expertise for safe and competent practice at the time of graduation.

Over the past years this Exit exam was developed with the faculty and the external examiners reports each year. Students who have completed all clinical requirements and passed all their assessments should also pass the Exit exam with a minimum grade of 70 out of 100 in order to graduate from the Dental College. The College has developed an overall protocol for the management of its assessment processes in line with international best practices.

A comprehensive approach that involves faculty and staff across all disciplines contributes to this integrated comprehensive multiform examination. The Exit exam consists of four components taken on 4-days apart: MCQ, MEQ, OSCE, and an oral examination. The first three parts represent 30% each, and the oral exam represents 10% of the total Exit exam grade of 100%. This high-stakes exam represents 40% of the students’ final graduation average grade of the BDS programme and the remaining 60% is obtained from the assessment grades in BDS 4 and 5 (30% each).

Almost all the academic staff at the College have been involved in setting up the exam, grading or invigilation pre- and post-COVID-19 pandemic. Engagement of faculty members in the process of exam preparation was essential as they focused on the real needs for didactic knowledge, clinical acumen, and communication skill needed in today’s excellent dentist. The MCQ, MEQ, and OSCE exam questions were reviewed carefully by a group of faculty some of whom are stream coordinators who are responsible for compiling and organizing the exam papers in order to make sure that questions were appropriate and measured the intended learning outcomes. The oral exam was conducted to test the students’ ability to communicate their knowledge through a discussion of cases presented by a panel of examiners from three different fields of dentistry.

### Description of the platforms used for the online assessment

#### The MCQ, MEQ, and OSCE

These three exams were delivered 3 days apart, using Blackboard ‘Bb’ (Version: SaaS deployment 3800.11.0-rel.34+ e7b8bbb) in combination with the Respondus Lockdown Browser (Version 2.0.6.06). This special browser prevents any activity on the PC other than the exam webpage itself. It also makes an audio-video recording of the student during the exam period using the PC’s camera. The Respondus Lockdown Browser has recently been used by the university as a security measure as well as part of student conduct code in online assessments.

Questions for the three ‘Bb exams’ (MCQ, MEQ, and OSCE) were vetted through online meetings on Teams, before they were uploaded to Bb by a small number of trained faculty.

The exam papers were made accessible to students automatically on the due date and time. Each exam started with an approximately 10 minutes ‘check-in’ process comprising a video recording of the student’s ID and environment. The exam countdown timer started only at the completion of the check-in process. A maximum of 10 minutes were allowed for ‘late arrival’ to ensure the exam integrity.

**Figure 1.**
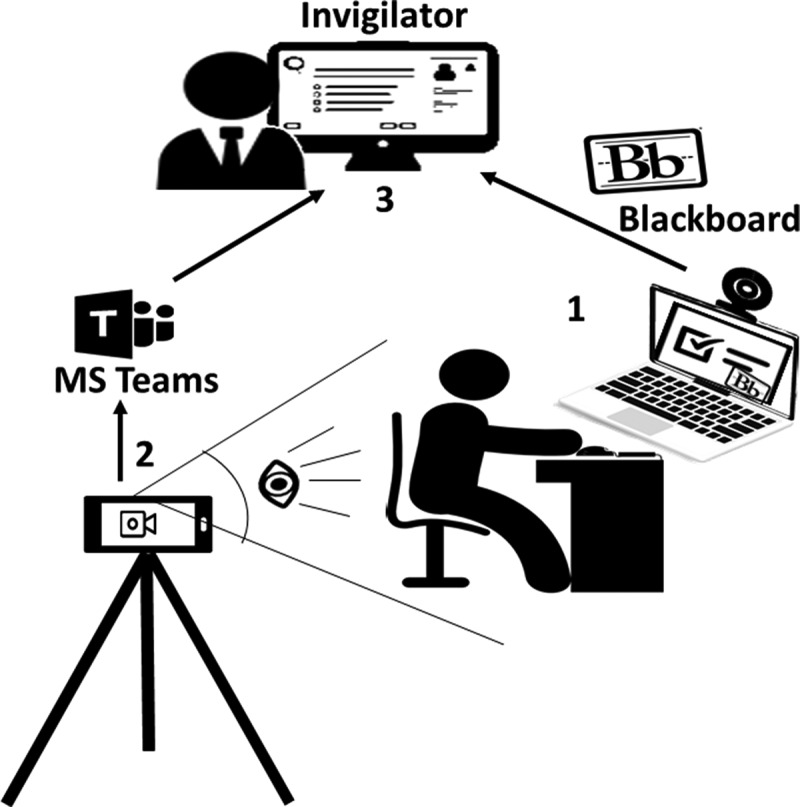
Online exam setup for the written parts of the examination (Modified Essay, Multiple-Choice and Objective Structured Clinical Examination Questions). 1: student performing online exam using Blackboard; 2: introducing invigilation method through using Microsoft Teams on mobile device (during the exam), and 3: completion of the invigilation process by reviewing the feedback from blackboard (following the exam).

A new method was put in place to invigilate the students and to provide instantaneous support in case of internet disconnection. The Respondus browser does not provide video recordings when the internet is disconnected. To overcome this limitation, an additional live communication tool was employed to provide the student with instant support in case of any internet disconnection, and to verify that the student did not attempt any cheating during the period of disconnection. Students were required to join Teams meetings with the invigilators in a ratio of 1 invigilator to 5 students. The meetings were conducted using smart phones operating the telecom data (4 G). This allowed for seamless connection with the invigilator in case of a drop of the Wi-Fi connectivity. The mobile phone was placed behind and to the side of the student (Figure 1) to allow the invigilator to verify if the student was looking at any material behind the PC/Laptop’s camera that was used for Bb recording.

Students who had lost access to the exam portal because of internet disconnection notified their respective invigilators instantly by telephone. They were placed in a one-to-one call on Teams with their respective invigilators to avoid disturbing other students in the invigilator’s group. The invigilator provided reassurance and some technical guidance to the students through a prepared troubleshooting guide. Once the internet connection was restored the invigilator made a call to the exam administrator in order to readmit the student back to the exam. At login, the student would be directed automatically to the same question they were working on when disconnected and would find the same remaining time (i.e. timer freezes at disconnection).

#### The oral exam

The oral exam, on the other hand, was conducted as an online meeting between the student and an examination panel using Microsoft Teams ‘Teams’ (Version 1.0).

**Figure 2.**
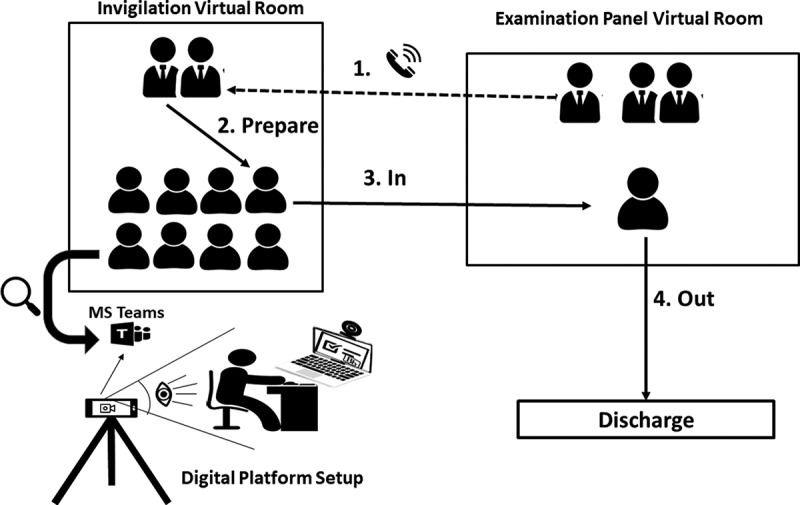
Online exam setup for the oral examination using Microsoft Teams. 1, 2, 3, and 4 sequence of events of admitting and discharging students during the online oral examination.

Invigilation for the oral exam was not applicable. However, to prevent communication between students who undertook the exam and those who were waiting to take it, the latter were ‘quarantined’ in Teams meetings (Figure 2). At the start of the exam, all students were placed in 5:1 meeting with the invigilators, using the students’ mobiles. Once the examination panel were ready to admit a new student, they would contact the invigilator to request the due student to join the panel meeting. The student would then receive a Teams call from the panel.

### Participants

An ethical approval was granted from the University of Sharjah prior to conducting the study (the ethical approval number: REC-20-06-22-01). The main investigator introduced the research project to the Bachelor of Dental Surgery (BDS) year 5 students and faculty members at University of Sharjah through an email invitation; then students were provided with information sheets and consent forms to take part voluntarily in the study to provide feedback on online assessment. As a result, 65 dental students (out of a total of 71) and 29 faculty members (out of a total of 33) were consented to participate in the study (Table 1).

**Table 1.** Mean satisfaction scores and standard deviation (SD) for participants’ gender and age group for the online assessment evaluation scales.

	Dental Students				Faculty Members	
	***N***	**Mean score (SD)**			***N***	**Mean score (SD)**
**Gender**				**Gender**		
Male	20	54.40 (13.91)		Male	20	48.95 (6.49)
Female	45	51.53 (10.87)		Female	9	44.78 (5.65)
**Age Group**				**Age Group**		
<23	4	65.50 (6.95) *		≤46	15	46.47 (7.45)
23–24	53	50.89 (11.86) *		>46	14	48.93 (5.14)
>24	8	56.00 (9.41)				
**Online Previous Experience**						
Yes	33	59.55 (9.48)♦				
No	32	45.06 (9.34)♦				

*, ♦ *p* < 0.05

### Online assessment surveys of stakeholders’ feedback

Evaluation of the online assessment consisted of 18 items for the dental students and 14 items for the faculty members that were rated on a five-point Likert scale from 1 (strongly disagree) to 5 (strongly agree). These items were designed to assess participants’ perception of their online assessment experience. The surveys comprised items related to the participants’ experience in online assessment, and satisfaction with the training, setting, administration, and environment. Two more open-ended questions were added to indicate challenges encountered and suggestions to improve online examination. An overall score for each individual was calculated by assigning a value of 1 for ‘strongly disagree’, 2 for ‘disagree’, 3 for ‘neutral’, 4 for ‘agree’, 5 for ‘strongly agree’ and these scores were then summed.

## Data analysis

All data were coded prior to data analysis. Tests of normality were performed where appropriate using normal probability plots [27] and equal variance tests [28]. Percentages of responses and the overall score for each individual were calculated. Mean values and standard deviations for each survey were calculated according to the method described by Field (2009) [29]. Students’ previous online experience was assigned to ‘yes’ and ‘no’ experience using a post-hoc median split and based on the continuous data of the survey score from question 2 in the survey ‘I have adequate previous experience in online learning system (e.g. Blackboard, MS Teams, Zoom, Google classroom … etc)’ [30]. Internal consistency reliability was assessed by Cronbach alpha (α) [31]. Pearson product-moment correlations were used to test the association between scores for each survey. Analysis of variance (ANOVA), with post-hoc analyses as appropriate, were used to assess the effect of differences in gender and age group on the outcome survey scores between groups and within the two surveys. For comparison, results related to the overall satisfaction with the online exam between students and faculty members was performed using an independent Student’s *t*-test. All data analyses were conducted using SPSS (version 25.0; SPSS Inc, Chicago, IL), and statistical significance for quantitative and categorical data was set at *p* < 0.05. Responses from the last two open-ended questions were analysed using a thematic analysis method as described by Braun and Clarke [32].

## Results

Data from 65 students (20 males and 45 females) and from 29 faculty members (20 males and 9 females) were available for analyses. Participation rates/response rates were: 92% for students and 88% for faculty members. Participants’ ages ranged from 22 to 26 years for dental students (mean age and standard deviation: 23.48 ± 0.85 years) and from 34 to 61 years for faculty members (mean age and standard deviation: 47.93 ± 7.78 years). Descriptive statistics of the effect of differences in gender, and age group on the outcome survey scores are presented in Table 1.

### Responses and scores of the two surveys

#### Students’ responses

The students’ online assessment survey had good internal reliability (Cronbach alpha of 0.89). Students’ responses to items within the scale ranged from 2% to 75% (Table 2). It was clear that the majority of participants responded with the ‘disagree category’ to 10 out of 18 items in this survey. ANOVA analysis showed a significant difference in scores between the three age groups, *p* = 0.036. A post-hoc test revealed that participants younger than 23 years old were found to have significantly higher satisfaction mean score compared to 23–24 age group, *p* = 0.034 (Table 1). No significant differences in the satisfaction score with online assessment were found between genders. For differences in level of previous online experience, an unpaired *t*-test revealed that the mean satisfaction survey score for the more experienced students was significantly higher than the one reported by the less experienced ones (*t* (62.98) = 6.20, *p* < 0.0001) (Table 1).

**Table 2.** Percentages for responses to items for the students’ online assessment Scale.

	Questions	%SD	%D	%N	%A	%SA
1)	Exam information and instructions were clearly communicated to me.	5	6	22	51	17
2)	I have adequate previous experience in online learning system (e.g. Blackboard, MS Teams, Zoom, Google classroom … etc).	23	26	14	32	5
3)	The training and the mock exams for the online exam were adequate to familiarize myself with the format of the exam.	6	20	14	37	23
4)	The exam training received made me feel less anxious about the online exam.	8	29	28	28	8
5)	I was satisfied with the accessibility and availability of the examination team to solve any issues during the exam.	5	8	25	45	19
6)	I was satisfied using Blackboard and MS Teams for online examination.	15	22	22	26	15
7)	I was satisfied with conducting different formats of the online exams.	12	31	34	15	8
8)	I am now comfortable to take an online exam.	15	25	25	25	11
9)	I was happy with the amount of time to prepare for the online exam compared with the paper-based exam.	23	19	32	15	11
10)	I was satisfied that my final grade reflected my performance in the exam.	14	20	29	29	8
11)	Online exams are more accessible than paper-based exams.	22	35	23	15	5
12)	Marking is more accurate, because computers do not suffer from human error.	15	23	42	17	3
13)	The technology used in online assessments is reliable.	12	15	40	28	5
14)	Online assessments favour some students more than others.	5	23	28	35	9
15)	The time given to complete the online exam was adequate.	42	25	23	8	3
16)	Preventing back-tracking in the online exam affected my performance negatively. (r)	75	11	9	3	2
17)	The oral online exam made me less anxious compared with the conventional oral exam.	5	5	26	37	28
18)	Overall, I was satisfied with this online exam.	14	19	40	23	9

**%SD **= Percentage of strongly disagree response, **%D **= Percentage of disagree response, **%N **= Percentage of neutral response, **%A **= Percentage of agree response, and **%SA **= Percentage of strongly agree response. (r) = reversed item

#### Faculty members’ responses

The faculty members’ online assessment survey used in the current study had good internal reliability (Cronbach alpha of 0.82). Faculty responses ranged from 0% to 66% (Table 3). It was clear that most participants responded with the ‘agree category’ to 10 out of 14 items in this survey. It was interesting to note that the majority of faculty members (83%) were satisfied with the innovative invigilation process used in the current study (Figure 1). No significant differences in the satisfaction score with online assessment were found between genders nor between different age groups.

**Table 3.** Percentages for responses to items for the faculty members online assessment Scale.

	Questions	%SD	%D	%N	%A	%SA
1)	The technology used for setting and administering the online exam was reliable.	3	0	3	55	38
2)	The method used for invigilating the exam (MS Teams and Blackboard) was reliable.	3	0	14	55	28
3)	It took me longer to prepare for the online exam as compared to the traditional one. (r)	7	14	14	52	14
4)	The online invigilation process used in this exam (MS Teams and Blackboard) was administrated smoothly.	3	0	10	66	21
5)	I feel that online examination is often frustrating because of technical problems. (r)	0	28	35	31	7
6)	I had to be more creative in terms of the resources used for the online examination. (r)	3	7	24	55	10
7)	It was more difficult for me to conduct the online multiformat examination than in the traditional setting. (r)	10	35	21	31	3
8)	I was satisfied with the way the online oral examination was conducted using MS Teams.	3	3	3	38	52
9)	The oral online exam made the student less anxious compared with the conventional oral exam.	7	10	55	14	14
10)	I was satisfied with the way the online OSCE examination was conducted using Blackboard.	3	7	21	45	24
11)	The OSCE online format exam assessed different knowledge/skills that would not be otherwise assessed with the written exam formats.	10	31	41	7	10
12)	The online exam has an advantage compared with the paper-based exam as it is more accessible to the students.	3	24	28	35	10
13)	Overall, I prefer the online grading compared with the paper-based exam grading.	14	17	21	31	17
14)	Overall, I was satisfied with this online exam.	3	0	10	62	24

**%SD **= Percentage of strongly disagree response, **%D **= Percentage of disagree response, **%N **= Percentage of neutral response, **%A **= Percentage of agree response, and **%SA **= Percentage of strongly agree response. (r) = reversed item

When comparing the mean satisfaction survey score for the question item: ‘Overall, I was satisfied with this online exam’ (question number 18 for students and 14 for faculty members) an unpaired t-test revealed that the mean satisfaction survey score for faculty members (mean = 4.03, SD = 1.07) was significantly higher than the one reported by students (mean = 2.86, SD = 0.82), (*t* (69.19) = −5.79, *p* < 0.0001).

#### Thematic analysis of students’ responses to the open-ended questions

Fifty-six students out of 65 (88%) responded to the question about the most significant challenges/difficulties they encountered during this online exam, and what they suggested to overcome these difficulties in future online examinations. Most of the students reported that the most significant challenge they faced during the online exam was the inadequate time in the MEQs paper and their limited ability in typing fast comparing to their handwriting. The second reported difficulty was their inability to go back to their answers (backtracking) during the MCQs paper as was the case in the previous paper-based exam. The blackboard system gives the faculty the option of allowing backtracking or not. However, for this high-stakes exam, it was decided not to allow backtracking during the MCQs exam to prevent cheating. It was also mentioned that sometimes the internet was slow or disconnected for a short time and it would be recommended to improve the internet in the future. Some students reported that online exam was stressful due to them being worried about getting disconnected, inadequate time in the essay, and no backtracking in the MCQs. Few students reported inconvenience noise from using MS teams for invigilation of group of students.

Regarding suggestions for future improvement of the online exam, most students indicated that the time for MEQs should be increased taking into consideration that some students are slow in typing. They also suggested that backtracking should always be allowed in the MCQs as long as students were monitored by an invigilation system. Furthermore, most students suggested decreasing the number of MEQs or choosing questions that would require shorter answers to help reducing the stress about exam time. Fewer students suggested removing the MEQs exam part altogether as they thought it needed time in typing. The students’ results obtained from the open-ended questions are summarized in Table 4.

**Table 4.** Thematic analysis of students’ responses to the open-ended questions.

#	Themes	Quotations
1	**Inadequate time in the MEQs online exam**	‘The MEQ exam was the most challenging and inadequate of the overall Exit exams, as well as the inability to backtrack through the other examinations’ ‘Insufficient time for MEQ exam and backtracking’ ‘Allow for more time in MEQ exams or format the questions to be more concise and shorter’
2	**Students’ typing skills in the MEQs online exam**	‘Time not all student type fast with the number of the questions were given in written exam’ ‘I was slow in typing for MEQ exam’ ‘Allow backtracking and no essay questions as some students are slow typers’
3	**Inability for backtracking in the MCQs**	‘No backtracking caused a huge issue for me as I am someone who likes to double check all her answers and if I don’t know an answer I like to leave it and come back to it at the end’ ‘That there was no backtracking in MCQs, Long MEQs and the time was not enough to solve all the questions in a correct way’ ‘Allow backtracking because it’s important and give more time and shorter questions for the MEQs’ ‘Allow back-tracking because definitely it’s needed in the MCQ’
4	**Technical issues due to internet quality**	‘The exam suddenly disconnected due to server issues’ ‘Being worried about getting disconnected’ ‘Typing was slow since it was lagging’ ‘Upgrade the system to avoid lagging while typing’ ‘Solve lagging and technical issues and increase time’
5	**Inconvenience noise from using MS teams**	‘Due to MS teams, there were a lot of background noise’ ‘Noise from MS teams’ ‘I think having the examination team online to help us was a great idea. I can’t think of another solution’

#### Thematic analysis of faculties’ responses to the open-ended questions

Twenty-four out of 29 (83%) faculty members responded to the question about the most significant challenges/difficulties they encountered during this online exam, and what they suggest to overcome these difficulties in future online examinations. Most faculty members reported that technical difficulties related the internet quality was the most significant challenge they faced during the online exam. The second reported difficulty by faculties was that online exam preparation took more time and efforts compared to a paper-based exam. It was also mentioned that grading online essay questions was more difficult and took longer time especially when many faculty members shared grading different questions in the online exam. One faculty reported that online exam was stress-inducing.

For future improvement of the online exam, most faculty members indicated that it was very important to have a robust IT support in the future. They have suggested to have a full time IT specialist available to assist in technical issues before, during and after the conduct of online exam to provide the necessary support for the preparation and grading of the exam. Moreover, they suggested to have more training sessions for faculty members in future exams. Furthermore, they suggested to allow more time for online exam preparation as it was too short this time due the sudden Covid-19 lockdown. Few faculty members suggested to improve the internet speed and have some diagnostic software regarding the effectiveness of the internet. The faculty members’ results obtained from the open-ended questions are summarized in Table 5.

**Table 5.** Thematic analysis of faculty members’ responses to the open-ended questions.

#	Themes	Quotations
1	**Technical difficulties related to the internet quality**	‘Technical difficulties specially the internet quality’ ‘Connectivity and lagging voice’ ‘Stability/Reliability of the internet connection at student’s end’
2	**The time and efforts in online exam preparation**	*‘a lot of preparation was needed compared to paper exam i.e. learning curve for faculty and some technical difficulties mainly from students’ side’* *‘needs more time to prepare, liable to problems of internet disconnections’*
3	**Grading online essay questions**	*‘The main challenge was that the system is not designed to allow more than one person to log in and correct the answers’* *‘Being able to correct the question simultaneously. Some technical issues related to smoothness of looking at questions and speed of the internet’*
4	**Importance of faculty training and IT support**	‘Repeat the excellent training provided by IT team’ ‘More formal training for the IT team on exam setting and marking using BB is required to overcome the above challenges’ ‘Have a full time IT support person continuously on standby mode strictly for our school’
5	**More time for online exam preparations**	‘Early preparation and upgrade the internet’

## Discussion

The assessment of various domains of competence in dental education requires multiple methods of assessment and constructive feedback in order to overcome the limitations of single assessment formats. Dental educators should be aware of the limitations of each method of assessment and its impact on learning [33]. The Commission on Dental Accreditation (CODA) standards state that ‘graduates must demonstrate the ability to self-assess, including the development of professional competencies and the demonstration of professional values and capacities associated with self-directed, lifelong learning’ [34].

We have demonstrated in this study that a comprehensive multi-format high-stakes exam could be run online uneventfully with an acceptable level of satisfaction by all stake holders. For example, students reported that taking the oral exam online reduced their levels of anxiety, an issue that has gained great significance in recent years [35–37]. They also scored other areas highly, including the availability of the exam team, the training (including the availability of mock exams) for the online delivery of the exam, and exam instructions and information. More importantly, being a newly introduced exam in a short period of time due to the unforeseen COVID-19 issue it would provide a good model for higher education institutes to follow and benefit from our experience especially during this time of uncertainty.

Our study showed that students with previous experience in online learning system were more satisfied with the online exam compared with those with less experience. This is understandable and expected as the former are more adept to the use of online systems than the latter. This finding is in agreement with a previous study by Marius et al. [38] which reported an increased degree of acceptance of e-assessment among students at a higher level in the medical school when compared with their younger peers. Another implication of this finding is that we cannot make an assumption that a certain cohort of students should have the same perception of e-learning and/or e-assessment based on the fact that they would most probably be of a similar age and belong to the same generation as they may have had different experience in terms of previous exposure to information technologies. Moreover, higher education institutes should prepare their graduates to the job market needs, part of which is maintaining life-long self-development that requires adequate online skills.

It was found in our study that younger students had higher satisfaction with online assessment than their older peers which may be explained by a higher ability of the younger students including online development skills [39].

Students were overall satisfied with the online exam, but they had some concerns regarding the time required to complete the MEQ part of the exam. Some students have attributed this to their perceived limited abilities to type with adequate speed. This might not have been the case as the amount of writing required to answer the majority of the MEQ part was limited and the faculty members made sure that the time required to answer the MEQ part was adequate as part of a routine practice when setting up an assessment. Further support to this explanation has been shown in a previous study that has found no significant differences could be identified due to the format in which the students had written their answers [40]. One possible reason for students’ perceptions of their low performance in the MEQ part could be attributed to the comprehensive use of the MCQ exam format at our college compared with the MEQ exam format and thus they were more accustomed to the former than the latter. Moreover, students were not happy about preventing them from back tracking (the ability to go back to previously answered questions) in the MCQ exam. Although this may have disadvantaged some students who prefer to leave questions unanswered to the end if they were uncertain of which option to choose and come back to them later, and also those students who always prefer to revise their answers at the completion of their exam, such a decision to prevent back tracking was taken by faculty members to limit the possibility of cheating and maintain the integrity of the exam. This was a sensible decision when weighing the risks of sacrificing the exam integrity with the benefits of not disadvantaging a few students especially with the use of e-assessment which has been reported to increase the possibility of facilitating cheating [41]. Furthermore, some students reported minor technical problems in terms of a slow internet and a noise from the usage of the MS teams as a platform for invigilating. These problems were overcome with the availability of IT and faculty members personnel during the whole exam to provide the required help and support to students who encountered any technical issues and the clear instructions and guidance that were given to them prior to the exam explaining to them what to do in the case of any technical problems.

Similarly, faculty were overall very satisfied with the online exam. However, they had similar concerns to students with regards to facing some technological issues and thus suggested to have more training and IT support in the future. Also, they reported that the online exam was more demanding when compared with the traditional paper-based exam in terms of the preparation time to complete the exam set up and grading of the exam. This was expected as the COVID-19 pandemic forced the introduction and implementation of a new online exam in a short period of time. The latter issues should significantly improve in the future as the online exam is phased in.

When students were compared with faculty members with regards to satisfaction with the online exam, faculty members were found to be more satisfied than students. Having run a complex multi-format new online exam in a short period of time and with minor issues, faculty members had full awareness of success of the conduct of the exam than students and thus, they were highly satisfied with the online exam. Students, on the other hand, were anxious of how they would perform in the online exam, being of a new format that they were not fully familiar with and of significant importance to their career progression and therefore, it is understandable that their satisfaction would be less than that of the faculty members.

It was reassuring to find that the outcome of the online Exit exam for the current cohort of dental students was similar to the previous conventional paper-based Exit exam of previous cohorts, although no formal analysis was carried out for such comparison. However, this will be part of a comprehensive further research in the near future.

## Conclusions

- A newly introduced multi-format online high-stakes exam was implemented successfully during the COVID-19 lockdown
- The majority of faculty members were satisfied with the innovative invigilation process using two online-platforms system (Blackboard and MS Teams)
- Students and faculty members were satisfied with the conduct of the new online exam with the faculty members being more satisfied than students
- Students with previous experience in online learning systems were more satisfied with the online exam than those with less experience
- The main issues raised by students’ satisfaction with the exam were: inadequacy of time for the MEQ part, prevention of back tracking in the MCQ part and minor technological issues
- The main issues raised by faculty members’ satisfaction with the exam were: increased time required to complete the exam setup and grading compared to the paper-based exam and minor technological issues.
